# Physical Activity and Natural Anti-VIP Antibodies: Potential Role in Breast and Prostate Cancer Therapy

**DOI:** 10.1371/journal.pone.0028304

**Published:** 2011-11-30

**Authors:** Milena Veljkovic, Violeta Dopsaj, Milivoj Dopsaj, Donald R. Branch, Nevena Veljkovic, Maria M. Sakarellos-Daitsiotis, Veljko Veljkovic, Sanja Glisic, Alfonso Colombatti

**Affiliations:** 1 Sveti Sava Hospital, Belgrade, Serbia; 2 Institute of Medical Biochemistry, Clinical Centre of Serbia, Belgrade, Serbia; 3 Faculty of Sport and Physical Education, University of Belgrade, Belgrade, Serbia; 4 Canadian Blood Services, Toronto, Ontario, Canada; 5 Center for Multidisciplinary Research, Institute of Nuclear Sciences VINCA, Belgrade, Serbia; 6 Department of Chemistry, Section of Organic Chemistry and Biochemistry, University of Ioannina, Ioannina, Greece; 7 Divisione di Oncologia Sperimentale, Centro di Riferimento Oncologico CRO-IRCCS, Aviano, Italy; Clermont Université, France

## Abstract

**Background:**

There is convincing evidence from numerous clinical and epidemiological studies that physical activity can reduce the risk for breast and prostate cancer. The biological mechanisms underlying this phenomenon remain elusive. Herein we suggest a role for naturally produced antibodies reactive with the vasoactive intestinal peptide (VIP) in the suppression of breast and prostate cancer, which we believe could offer a possible molecular mechanism underlying control of these cancers by physical exercise.

**Methodology and Results:**

We found that sera from individuals having breast and prostate cancers have decreased titers of VIP natural antibodies as demonstrated by a lower reactivity against peptide NTM1, having similar informational and structural properties as VIP. In contrast, sera collected from elite athletes, exhibited titers of natural NTM1-reactive antibodies that are significantly increased, suggesting that physical activity boosts production of these antibodies.

**Significance:**

Presented results suggest that physical exercise stimulates production of natural anti-VIP antibodies and likely results in suppression of VIP. This, in turn, may play a protective role against breast and prostate cancers. Physical exercise should be further investigated as a potential tool in the treatment of these diseases.

## Introduction

The vasoactive intestinal peptide (VIP) is a pleiotropic peptide important in many physiologic functions, including glucose homeostasis, neuroprotection, memory, gut function, modulation of the immune system and circadian function. In addition, there are numerous evidences that (VIP) and its receptors, which are highly expressed in breast tumor cells [1],[2],[3],[4],[5],[6],[7],[8], play an important role in the pathogenesis of breast cancer. VIP functions as an autocrine growth factor [8],[9] and regulates proliferation, survival, and differentiation in human breast cancer cells [10]; it has proangiogenic functions [11] in breast cancer. VIP also induces transactivation of epidermal growth factor receptor (EGFR) and human epidermal growth factor-2 receptor (HER2) and increases expression of *c-fos*, *c-jun* and *c-myc* oncogenes [2],[12],[13]. There also is mounting evidence that VIP is involved in the pathogenesis of prostate cancer. VIP increases the expression of the major angiogenic factor VEGF [14] and acts as a proangiogenic factor [15],[16],[17]. VIP increases neuroendocrine differentiation [18] and stimulates interleukin-6 production [19] and prostate-specific antigen (PSA) secretion in prostate cancer [20]. In addition, VIP stimulates HER2 transphosphorylation in androgen-independent prostate cancer cells [17], stimulates their invasive capacity [21] and contributes to prostate cancer pathogenesis by induction of malignant transformation [22]. VIP antagonists suppress the release of prostate-specific antigen (PSA) [23] and inhibit growth of breast and prostate cancer cells [24],[25],[26],[27],[28],[29].

Taken together, these observations strongly support the notion that VIP plays an important role in breast and prostate cancer pathogenesis and suggests that elevated concentrations of VIP in the circulation may represent a risk factor for these cancer types.

VIP is elevated in plasma after aerobic exercise [30],31[31],[32],[33],[34],[35],[36] suggesting that physical activity represents a potential stimulus for VIP autoantibody formation [37],[38]. Because of the immunomodulatory and neuromodulatory activities of VIP, circulating levels of this peptide are under tight control and natural anti VIP autoantibodies are potent modifiers of its biological actions and important regulators of its circulating level [39],[40],[41],[42],[43]. However, the antigenic stimulus leading to the formation of these autoantibodies has not been identified yet. It was previously shown that structural and informational similarity, represented by the informational spectrum frequencies, is essential for the immunological cross-reactivity between VIP and HIV-1 gp120 [44],[45],[46],[47]. This analysis revealed that peptide FTDNAKTI (NTM1) represented the shortest gp120-derived peptide resembling informational and structural properties of VIP [48].

The present study was carried out in order to compare reactivity with peptide NTM1 of sera collected from individuals with breast and prostate cancer and sera from elite athletes. Obtained results showed a significant difference in NTM1 reactivity of sera from elite athletes, healthy controls and cancer patients.

## Results

**Figure 1:**
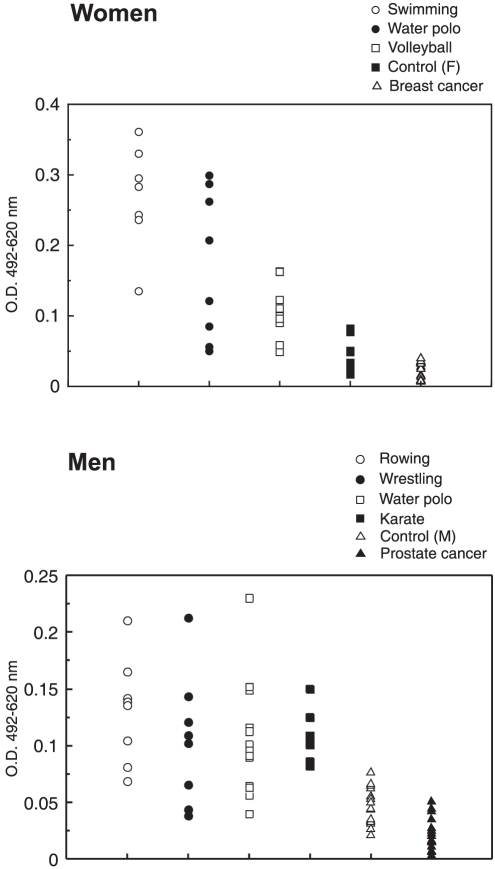
Results of ELISA. The absorbance values (OD) obtained for sera of cancer patients, athletes and healthy control subjects with peptide NTM1. Antibodies recognizing peptide NTM1 are significantly more prevalent in serum samples from athletes compared to control subjects (swimming P(2.9e-06), water polo P(0.0001), volleyball P(8.1e-07), rowing P(1.8e-06), wrestling P(0.0009), and karate P(3.2e-07); Mann-Whitney test). The absorbance values for sera from cancer patients are significantly lower in comparison with the values obtained for control sera (P(0.02) and P(3.7e-05) in breast and prostate cancer, respectively; Mann-Whitney test).

Reactivity of sera collected from subjects with breast and prostate cancer with peptide (NTM1s)_4_-SOC_4_ was determined by the ELISA immunoassay (Figure 1 and Table 1). Statistical analysis revealed that this reactivity in sera from cancer patients was significantly lower in comparison with the reactivity of control sera P(0.02) and P(3.7e-05) in breast and prostate cancer, respectively). In Table 2 is given the total IgG content determined for breast and prostate cancer patients.

**Table 1:** Comparison of the reactivity with peptide NTM1 of sera from cancer patients, athletes and healthy control subjects.

Tested population	Number of subjects	Gender	O.D. (mean)	O.D. (± s.d.)

***Cancer patients***				
Breast cancer	15	F	0.021	0.011
Prostate cancer	17	M	0.022	0.014
***Athletes***				
Swimming	7	F	0.269	0.074
Water polo	8	F	0.171	0.105
Volleyball	13	F	0.104	0.033
Rowing	8	M	0.131	0.046
Wrestling	9	M	0.106	0.054
Karate	9	M	0.108	0.020
***Controls***				
	17	F	0.041	0.025
	17	M	0.046	0.016

**Table 2:** Data for investigated breast and prostate cancer patients and the absorbance values obtained for their sera with peptide NTM1.

Patient	Type of cancer	Disease stage	Age	Tumor marker*	Total IgG [g/l]	NTM1 (OD)
P1	breast	III	42	49.4	13.9	0,016
P2	breast	IV	59	104.9	12.9	0,016
P3	breast	IV	61	74.7	9.2	0,032
P4	breast	II	39	43.4	11.5	0,006
P5	breast	IV	49	99.8	9.8	0,028
P6	breast	IV	47	172	11.2	0,024
P7	breast	IV	55	98.7	11.4	0,035
P8	breast	II	36	36.4	6.0	0,034
P9	breast	IV	49	2533	12.6	0,032
P10	breast	III	51	54.5	8.0	0,036
P11	breast	IV	52	366	16.4	0,014
P12	breast	III	56	64	9.0	0,008
P13	breast	IV	63	199	10.7	0,025
P51	breast	IV	59	1014	13.0	0,009
P207	breast	IV	64	186	12.0	0,008
P1	prostate	IV	60	238,0	11,4	0,020
P2	prostate	III	56	31,1	9,95	0,016
P3	prostate	IV	62	47,0	12,4	0,025
P9	prostate	II	48	17,1	16,9	0,027
P69	prostate	III	59	40,5	8,41	0,007
P105	prostate	II	50	18,3	14,2	0,015
P179	prostate	III	62	22,2	11,4	0,023
P182	prostate	II	48	12,7	14,7	0,020
P8	prostate	IV	66	150,0	16,4	0,016
P94	prostate	IV	72	249,9	16,5	0,044
P76	prostate	III	58	32,6	11,4	0,050
P356	prostate	II	52	13,5	12,1	0,011
P344	prostate	IV	69	49,5	14,9	0,035
P55	prostate	IV	64	58,6	14,28	0,042
P120	prostate	IV	68	154,0	16,3	0,006
P222	prostate	III	54	21,9	13,6	0,011
P318	prostate	IV	70	280,0	10,1	0,003

*Tumor markers: breast cancer (CA15-3); prostate cancer (PSA).

In Figure 1 and Table 1 are presented the results of ELISA testing of sera collected from elite athletes. Analysis showed that (NTM1s)_4_-SOC_4_-reactivity of sera from elite athletes was highly significantly different (i) from reactivity of sera collected from healthy, sedentary controls (for female: swimming P(2.9e-06), water polo P(0.0001), volleyball P(8.1e-07), all female elite athletes P(2e-6); for male: rowing P(1.8e-06), wrestling P(0.0009), karate P(3.2e-07), all male elite athletes P(2e-6)), and (ii) from sera from cancer patients (for breast cancer: swimming P(1.2e-05), water polo P(4.1e-6), volleyball P(7.1e-06), all female elite athletes P(2e-6); for prostate cancer: rowing P(1.8e-06), wrestling P(1.2e-5), karate P(6.4e-07), all female elite athletes (2e-9).

## Discussion

There is now strong and consistent evidence from several studies conducted worldwide that regular physical activity reduces breast cancer risk by 20% to 30% and that a dose–response effect exists [49]. Long-term athletic training during the college and pre-college years lowers the risk of breast cancer throughout the life span [50],[51],[52]. For tertiary cancer prevention, observational studies suggest that breast cancer survivors performing exercise (e.g., 2–3 h of brisk walking/week) have a 40–67% reduction in breast cancer recurrence and all-cause mortality compared with inactive survivors [53],[54],[55]. Similar data were reported for prostate cancer where an average risk reduction ranged from 10%–30% [56],[57]. Recently, the evaluation of physical activity in relationship to prostate cancer mortality among 2,750 men diagnosed with prostate cancer showed that a modest amount of vigorous activity, such as biking, tennis, jogging, or swimming for 3 hours a week, may substantially improve prostate cancer-specific survival [58],[59].

However, the potential biologic mechanisms through which physical activity may decrease the risk of breast and prostate cancer are still elusive. Several putative etiologic pathways, including those involving steroid hormones, chronic inflammation, growth factors, lymphokines and insulin resistance were suggested (reviewed in Ref. [49]). On the other hand, there are mounting evidences that VIP pathway plays an important role in pathogenesis of breast and prostate cancer, indicating that elevated concentration of VIP, a facilitator of breast and prostate cancer, in the circulation may contribute to these diseases and that suppression of this peptide could have positive effect [24],[25],[26],[27],[28],[29].

The level of circulating VIP is controlled by natural anti-VIP antibodies [39],[40],[41],[42],[43] and we hypothesized that these suppressive antibodies could contribute to a better control of breast and prostate cancer and that lack of these antibodies could contribute to the progression of these diseases. In order to test this assumption we compared reactivity with peptide NTM1 of sera collected from cancer patients and healthy control. Results given in Table 1 and Figure 1 show that this reactivity is significantly lower in cancer patients in comparison with healthy control subjects.

Paul and Said showed that natural anti-VIP antibodies were present in plasma from 29.6% of healthy human subjects who habitually performed aerobic muscular exercise (running, cycling, swimming, aerobic dancing, and/or weight training, 3 or more workouts per week for a year or more prior to study entry), compared to 2.3% of healthy subjects who did not [37]. Herein, presented results show that a significantly higher percentage of trained athletes had increased titers of NTM1 reactive antibodies, 23 out of 28 (82.1%) females and 23 out of 26 (88.5%) males compared to non-athletes. This finding expanded our recent report on male water polo elite athletes [38] and strongly supported the suggestion that physical exercise represents a stimulus of the immune system which boosts production of natural VIP/NTM1-reactive antibodies.

The total IgG content in all cancer patients (11±2.7 mg/ml) and in elite athletes (11.2±2.9 mg/ml) was in the normal range (7–16 mg/ml) and comparable with normal values suggesting that that difference in the different age distribution of the athletic subjects (18–26) and cancer patients (56±3.9) did not significantly affect global function of the immune system, allowing comparison of immunological data obtained for these two studied groups. Thus, the reduced levels of natural antibodies recognizing peptide NTM1 in breast and prostate patients was specific and may contribute to disease progression.

The antigenic stimulus for the formation of natural VIP/NTM1-reactive antibodies could not be identified from our studies; however, several studies have demonstrated that acute exercise is associated with increased plasma levels of VIP [30],[31],[32],[33],[34],[35],[36]. It is thus plausible to theorize that these antibodies may have been produced in response to an increase in VIP levels during exercise, providing increased antigenic stimulus. In line with this hypothesis is that physical activity provides a potential source of the natural anti-VIP/NTM antibodies that could contribute to the control of breast and prostate cancer.

In order to keep the conclusions derived from this study under its real extent, it is necessary take into account that elite athletes are different from sedentary and even from physically active subjects, not just because of the exercise routine, but mainly for genetic predisposition allowing remarkable physiological and biological indexes. For this reason, we can't exclude the possibility that these specific genetic makeup of elite athletes could also partially contribute to their increased production of natural VIP/NTM1-reactive antibodies.

Herein we suggest a possible role for naturally produced antibodies reacting with peptides VIP and NTM1 in the control of breast and prostate cancer, which we believe could offer a possible molecular mechanism underlying positive effects of physical exercise in these cancers. Presented results strongly suggest further research of physical exercise as an important natural approach against breast and prostate cancer. Because little is known about the optimal level of exercise in combating of these cancers, variations in the mode, intensity, duration, and frequency of exercise prescriptions also requires future research.

## Materials and Methods

### Ethics statement

Informed consent and local ethics committee approval was obtained for these human studies. All patients provided written informed consent. The Ethical Committee for Clinical Trials of the Faculty of Pharmacy in Belgrade is responsible for the ethical conduct of this study.

### Human subjects

#### Cancer patients

Sera samples were collected from 15 subjects with breast and 17 subjects with prostate cancer (disease stage, values of tumor markers and the age are given in Table 2). Sera were collected immediately after confirmed diagnosis and before the start of any therapeutic intervention.

#### Athletic subjects

Sera samples were collected from 54 Serbian elite international athletes (26 males and 28 females) engaged in the following types of sports activities: wrestling, water polo, rowing, karate, kick boxing, swimming and volleyball. Female and male elite athletes were engaged in the sport training in average 9 and 11 years, respectively. All athletes were tested at the end of basic preparatory mezocycle (a phase of training with duration of between 4–6 weeks) in a period of recuperative microcycle (shorter training period of about 7–10 days for active recuperation of the athletes) [60]. We expected that in this latter period, production of natural antibodies which clear VIP from circulation should have reached higher levels.

#### Controls

Sera samples were collected from 34 healthy (HIV-negative) individuals (17 males and 17 females) doing sedentary computer work 6–8 h a day and who were not performing regular physical exercise (healthy, sedentary subjects). The ages of all controls (18–26) matched the athletic subjects and there were no risk factors that would affect the immune system (cigarette smoke, alcohol consumption, use of medications, chronic diseases, etc).

### Peptide conjugates synthesis

The synthesis of the (NTM1s)_4_-SOC_4_ conjugate was carried out manually by stepwise solid phase peptide synthesis using the Boc-Gly-OCH_2_-Pam resin (1 g, 0.25 mmol/g capacity). Sequential oligopeptide carrier (SOC_4_), formed by the repetitive Lys-Aib-Gly, is applied to display analyzed peptides. The synthetic procedure starts with the step-by-step couplings of the protected residues (Boc/Bzl) corresponding to the SOC_4_ carrier to the resin. Lysine was introduced as Boc-Lys(Fmoc)-OH. After removal of the Fmoc protective groups from the Lys-N^ε^H_2_ groups by 20% piperidine in dimethylformamide, synthesis of the epitope FTDNAKTI was carried out (Boc/Bzl) by the simultaneous attachment of each residue in four copies.

### ELISA

ELISA was performed with peptide (NTM1s)_4_-SOC_4_ by the following procedure: polystyrene microtest plates (Sarstedt, Germany) were incubated overnight at 4°C with 100 µl of peptides (0.5 µg/well) diluted in carbonate buffer, pH 9.6. Plates were washed with phosphate-buffered saline (PBS)–0.05% Tween and non-specific sites were blocked with 200 μl PBS containing 5% bovine serum albumin (BSA) for 2 h at room temperature. After six washings, serum specimens were added to the wells (100 µl/well). Sera were diluted 1∶100 in 5% BSA in PBS. Plates were incubated for 3 h at room temperature. After six washings with PBS–0.05% Tween, 100 µl of goat anti-human IgG alkaline phoshatase-conjugated antibodies (Sigma), diluted 1∶2500 were added and the plates were incubated for 30 minutes at room temperature. After six washings, pNPP (p-Nitrophenyl Phosphate) substrate was added and the absorbance (OD) measured at 492–620 nm after 15 minutes. Each sample was tested independently twice and the mean values are reported.

### Statistical analysis

The significance of the differences in O.D. values for individuals with breast and prostate cancer, elite athletes and control subjects was calculated by the non-parametrical Kruskal-Wallis. The two groups are unpaired with uneven variance and therefore they were considered as part of a two-tailed, heteroscedastic matrix. For each comparison, the level of significance p for a directional test is given.
